# Insights into the protein ubiquitinome in the host‒pathogen interplay during *Mycobacterium tuberculosis* infection

**DOI:** 10.3389/fmolb.2025.1613454

**Published:** 2025-08-26

**Authors:** Qishun Feng, Qiao Lin, Guoxin Huang, Siqi Li, Yating Xu, Taosheng Ye, Guoliang Zhang

**Affiliations:** ^1^ National Clinical Research Center for Infectious Diseases, Shenzhen Third People’s Hospital, Shenzhen, China; ^2^ The Baoan People’s Hospital of Shenzhen, The Second Affiliated Hospital of Shenzhen University, Shenzhen, China; ^3^ Shenzhen Hospital of Guangzhou University of Chinese Medicine (Futian), Shenzhen, China

**Keywords:** *Mycobacterium tuberculosis*, macrophage, host-pathogen interaction, ubiquitinome, immune response

## Abstract

*Mycobacterium tuberculosis* (Mtb) is the causative agent of tuberculosis capable of manipulating and circumventing the host’s immune system to establish infection. Ubiquitination plays a crucial role in the host’s response to pathogens; however, the global alterations in protein ubiquitination during Mtb infection remain poorly understood. To elucidate the regulatory roles of ubiquitination in the immune response to Mtb, we investigated the ubiquitome of human macrophages following Mtb infection. In our study, we identified a total of 1,618 proteins exhibiting altered ubiquitination levels, with 1,182 lysine-ubiquitination sites in 828 proteins showing increased ubiquitination and 1,077 sites in 790 proteins displaying decreased ubiquitination. Bioinformatics analyses revealed that most proteins involved in the immune response were upregulated, including those associated with autophagy, lysosome, the NF-κB signaling pathway, necroptosis, and ferroptosis. Furthermore, the ubiquitination levels of numerous proteins involved in conserved physiological processes, such as ribosome biogenesis, spliceosome function, nucleocytoplasmic transport, and mRNA surveillance, were also altered, suggesting that these pathways may be regulated by ubiquitination during Mtb infection. The extensive pool of ubiquitinated proteins and sites identified in this study will serve as a valuable resource for understanding the regulatory mechanisms of the ubiquitination system in immune responses during Mtb infection.

## Introduction

*Mycobacterium tuberculosis* (Mtb), the causative agent of tuberculosis (TB), remains the leading infectious agent responsible for human mortality (Feng et al., 2024a). According to the World Health Organization (WHO) (Goletti et al., 2024), Mtb was responsible for 1.25 million deaths and approximately 10.8 million new infections globally in 2023. The emergence of drug-resistant Mtb strains has rendered existing TB medications ineffective (Feng et al., 2024b). Consequently, there is an urgent need to develop new TB therapeutics based on innovative strategies and targets that effectively combat drug-resistant TB. As an intracellular pathogen, Mtb can persist within host macrophages by manipulating host signaling pathways and cellular processes, including innate immune signaling pathways and phagocytosis (Liu et al., 2017).

The strength of the host’s immune system significantly influences the outcomes of Mtb infections. Host resistance to TB relies on the activation of both adaptive and innate immune responses. Macrophages, as key innate immune cells, play a critical role in mediating resistance to TB (McClean and Tobin, 2016). They recognize invading pathogens, activate bactericidal mechanisms, and coordinate the overall immune response. Autophagy and macrophage-associated cell death mechanisms, such as pyroptosis, ferroptosis, and apoptosis, are essential for managing harmful bacterial infections (Schorey and Schlesinger, 2016). However, Mtb can exit and disseminate from the infected cell through necrosis and necroptosis, thereby facilitating the spread of the infection (Schorey and Schlesinger, 2016). Mtb can also evade the host’s immune response, leading to the development of latent tuberculosis infection (LTBI) or active tuberculosis (ATB). By secreting early anti-apoptotic proteins such as NouG, PknE, and SecA2, Mtb can inhibit macrophage apoptosis and enhance its survival within these cells. Furthermore, by binding to Toll-like receptor 2 (TLR2) on the surface of macrophages, Mtb activates the NF-κB pathway, which upregulates the production of the anti-apoptotic protein BCL2, thereby preventing macrophage apoptosis (Zhuang et al., 2024).

Ubiquitination modifies proteins and lipids post-translationally, regulating their activation, signaling capacity, molecular stability, and deactivation in response to danger signals (Liu et al., 2016). It also directly targets infectious particles for destruction in phagolysosomes and autophagolysosomes (Sharma et al., 2018; Chen et al., 2019). Ubiquitination plays a critical role in controlling Mtb by modulating the MAPK pathway, apoptosis, xenophagy, and inhibiting inflammation (Campos et al., 2022). Despite several studies reporting the role of ubiquitination in restricting Mtb infection, a comprehensive understanding of this significant post-translational modification in the host immune response to Mtb infection remains lacking.

In this study, we investigated the protein ubiquitination status of human macrophages following Mtb infection. By utilizing immunological affinity purification, liquid chromatography-tandem mass spectrometry (LC-MS/MS), and protein functional categorization analysis, we identified 1,618 proteins with altered ubiquitination levels. Subsequent analyses revealed that the ubiquitination system can modulate several immune response pathways, including autophagy, lysosomal function, the NF-κB signaling pathway, necroptosis, and ferroptosis. Furthermore, ubiquitination regulates a variety of cellular functions in response to Mtb infection, such as ribosomal activity, spliceosomal function, mRNA surveillance pathways, and nucleocytoplasmic transport. Our findings provide new insights into the role of the ubiquitination system in Mtb-host interactions.

## Materials and methods

### Cell culture

The human monocytic cell line THP-1 was obtained from the National Collection of Authenticated Cell Cultures in China and maintained in RPMI 1640 medium (Gibco, 11,875,093) supplemented with 10% fetal bovine serum (Gibco, 10091148), 0.05 mM 2-mercaptoethanol (Sigma, M3148), 1 mM sodium pyruvate (Gibco, 11360–070), and 1% penicillin-streptomycin (Gibco, 15140122). The cells were cultured in a humidified incubator at 37 °C with 5% CO_2_. Antibiotics were not used during infection experiments.

### Bacterial culture

*Mycobacterium tuberculosis* H37Rv was cultured in Middlebrook 7H9 broth (BD Biosciences, 271310), supplemented with 10% Oleic Acid-Dextrose-Catalase (OADC) (BD Biosciences, 212240), 0.5% glycerol, and 0.05% Tween 80 at 37 °C to reach the mid-logarithmic phase (optical density at 600 nm [OD600] ≈ 0.8). The cultures were harvested, resuspended in phosphate-buffered saline (PBS) containing 0.05% Tween 20% and 25% glycerol, and stored at −80 °C. One vial of the stock was thawed to determine the colony-forming units (CFU) per milliliter. On the day of infection, the mycobacteria were thawed, washed, and sonicated prior to use.

### Sample preparation

THP-1 cells were seeded at a density of 1 × 10^7^ cells per well in a 10 cm cell culture dish, and differentiated with phorbol 12-myristate 13-acetate (PMA; Sigma, P8139) at a concentration of 100 ng/mL for 24 h. Following differentiation, the cells were allowed to rest in media devoid of PMA for an additional 24 h. Subsequently, the cells were infected with Mtb strain H37Rv at a multiplicity of infection (MOI) of 10 for 4 h (Budzik et al., 2020). Subsequently, the cells were washed three times with pre-warmed sterile phosphate-buffered saline (PBS) to remove extracellular bacteria, and cultured in RPMI 1640 medium at 37 °C with 5% CO_2_. After 3 h, the cells were harvested and lysed in RIPA lysis buffer containing 1% protease inhibitor cocktail and 50 μM PR-619, incubated on ice for 30 min. The protein concentration of the resultant lysates was determined using a bicinchoninic acid protein assay kit (Beyotime, P0010S).

The protein sample was mixed with one volume of pre-cooled acetone, and then combined with four volumes of pre-cooled acetone, followed by precipitation at −20 °C for 2 h. The precipitate was washed 2 to 3 times with the pre-cooled acetone. The protein sample was then redissolved in 200 mM TEAB and ultrasonically dispersed. Trypsin was added at 1:50 trypsin-to-protein mass ratio for overnight digestion. The sample was reduced with 5 mM dithiothreitol for 30 min at 56 °C and alkylated with 11 mM iodoacetamide for 15 min at room temperature in the dark. Finally, the peptides were desalted by using a Strata X solid-phase extraction (SPE) column.

### Affinity enrichment of ubiquitinated peptides

Dissolve the peptides in IP buffer solution (100 mM NaCl, 1 mM EDTA, 50 mM Tris-HCl, 0.5% NP-40, pH 8.0), transfer the supernatant to the pre-washed resin (PTM1104, from Hangzhou Jingjie Biotechnology Co). After incubation, the resin was washed four times with IP buffer solution and twice with deionised water. Finally, the resin-bound peptides were eluted using 0.1% trifluoroacetic acid eluent for a total of three times, and the eluent was collected and freeze-dried under vacuum. After extraction and desalting according to the C18 ZipTips instructions, the solution was freeze-dried under vacuum for liquid-quantity-mass spectrometry (LQM) analysis.

### Mass spectrometry (MS)

The peptide segments were dissolved in mobile phase A for liquid chromatography and subsequently separated using the Vanquish Neo ultra-high performance liquid chromatography (UHPLC) system. Mobile phase A consists of an aqueous solution containing 0.1% formic acid, while mobile phase B is an aqueous solution containing 0.1% formic acid and 80% acetonitrile. The liquid chromatography gradient settings are as follows: 0–0.5 min, 4% B; 0.5–0.6 min, 4%–8% B; 0.6–13.6 min, 8%–22.5% B; 13.6–20.5 min, 22.5%–35% B; 20.5–20.9 min, 35%–55% B; 20.9–21.4 min, 55%–99% B; and 21.4–22.6 min, 99% B, with a flow rate maintained at 400 nL/min. The peptide segments are separated using the ultra-high performance liquid chromatography system and subsequently injected into the NSI ion source for ionization, followed by analysis in the Orbitrap Astral mass spectrometer. The ion source voltage is set at 1900 V, with the parent ions of the peptides detected and analyzed using the Orbitrap detector, while the secondary fragment ions are detected and analyzed using the Astral detector. The primary mass spectrometry scan range is set from 380 to 980 m/z, with a scan resolution of 240,000. The secondary mass spectrometry scan range has a fixed starting point at 150 m/z, with a secondary scan resolution of 80,000. The data acquisition mode employs the data-independent acquisition (DIA) procedure, in which, following the primary scan, peptide ions within multiple consecutive m/z windows are introduced into the HCD collision cell and fragmented using 25% collision energy, followed sequentially by secondary mass spectrometry analysis. To enhance the effective utilization of the mass spectrometer, the automatic gain control (AGC) is set to 500%, and the maximum injection time is set to 3 m.

### Database search and quantification

The resulting MS/MS data were processed using the DIA-NN search engine (version 1.8). Tandem mass spectra were searched against the XA06430B1DA_DPUb.fasta database, which contains 24,475 entries, concatenated with a reverse decoy database. Trypsin/P was specified as the cleavage enzyme, allowing for up to two missed cleavages. Carbamidomethylation on cysteine residues was specified as a fixed modification, while ubiquitylation on lysine residues was specified as a variable modification. The false discovery rate (FDR) was adjusted to be less than 1%.

The raw LC-MS datasets were initially searched against the database and converted into matrices containing the intensity of peptides across samples. The intensities of modified peptides (I) were centralized and transformed into relative quantitative values (R) using the following formula: R_ij = I_ij/Mean (I_j), where i denotes the sample and j denotes the modified peptide. To assess the statistical significance of the differences between groups, the Student’s t-test was performed on the relative quantitative values of each protein from the two sample groups. Proteins with a p-value <0.05 and a fold change >1.5 were considered significantly upregulated, while those with a p-value <0.05 and a fold change <1.5 were regarded as significantly downregulated. The mass spectrometry proteomics data have been deposited to the ProteomeXchange Consortium *via* the PRIDE (Perez-Riverol et al., 2025) partner repository with the dataset identifier PXD063016.

### Bioinformatic analysis

#### GO analysis

The Gene Ontology (GO) annotation of the ubiquitination proteome was obtained from the UniProt-GOA database. The GO annotation process involves utilizing the eggnog-mapper software to extract GO IDs from the identified proteins, based on the EggNOG database. Subsequently, functional classification annotation analysis is conducted on the proteins, categorizing them according to cellular components, molecular functions, and biological processes.

#### KEGG analysis

The Kyoto Encyclopedia of Genes and Genomes (KEGG) database was utilized to annotate the protein pathways. KEGG terms with a significance level of p-value <0.05 were deemed to be enriched. Additionally, the annotation of protein structural domains for the identified proteins was conducted using the Pfam database and the associated PfamScan tool.

#### Clustering analysis

Based on Fisher’s exact test p-value obtained from the enrichment analysis, the hierarchical clustering method is employed to group related functions across different Q groups and visualize them in a heatmap. The horizontal axis of the heatmap represents the various Q groups, while the vertical axis displays the related functions enriched by differentially expressed modified proteins, including GO terms, KEGG pathways, and protein domains. If a corresponding image for the analysis description is absent, the data cannot be mapped for that category. The color blocks corresponding to the enrichment functional descriptions of different Q groups and differentially expressed modified proteins indicate the level of enrichment significance. Blue signifies high enrichment significance, while a gradient of blue to white indicates low enrichment significance. An asterisk (*) denotes a p-value <0.05, two asterisks (**) indicate a p-value <0.01, and three asterisks (***) represent a p-value <0.001.

#### Protein interaction network analysis

The database IDs of differentially modified proteins or their sequences, screened based on a fold change of 1.5 in the comparison group, were analyzed in conjunction with the STRING protein-protein interaction network database. The protein-protein interaction relationships of these differentially modified proteins were extracted with a confidence score greater than 0.7, indicating high confidence. Subsequently, the resulting protein-protein interaction network was visualized using the R package ‘visNetwork’. As illustrated in the figure, the circles represent differentially modified proteins, with varying colors denoting their differential expression: blue for downregulated modified proteins, red for upregulated modified proteins, and yellow for modified proteins containing both up- and downregulated modification sites. The size of each circle corresponds to the number of interacting proteins. To clearly illustrate the interactions among proteins, we selected the top 50 proteins exhibiting the most tightly interconnected relationships to construct the protein-protein interaction network.

## Results

### Quantitative analysis of the THP-1 ubiquitome after *Mycobacterium tuberculosis* infection

**FIGURE 1:**
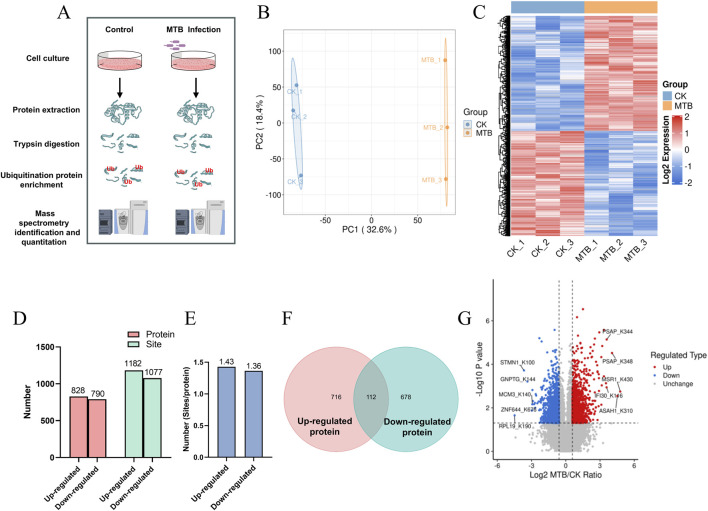
Ubiquitome profiling of Mtb-infected human macrophage THP-1. **(A)** Workflow for quantitative profiling of the ubiquitome in THP-1 macrophages infected with Mtb. **(B)** PCA shows the ubiquitome profiles. Each dot indicates one biological replicate. **(C)** Hierarchical clustering analysis and a heatmap of the proteins with differentially changed ubiquitination. **(D)** The number of proteins and ubiquitination sites that are significantly up- or downregulated after Mtb infection (fold-change >1.5, p < 0.05). **(E)** The average number of ubiquitination sites per protein. **(F)** Venn diagram showing the overlap between proteins with upregulated ubiquitination and downregulated ubiquitination after Mtb infection. **(G)** Volcano plot shows the proteins with changed ubiquitination after Mtb infection (fold-change >1.5, p < 0.05).

To examine the diversity and relative abundance of ubiquitinated proteins induced by Mtb, we infected human macrophage THP-1 cells with Mtb H37Rv for 7 h at a multiplicity of infection (MOI) of 10:1. The experimental workflow for our study is illustrated in Figure 1A. Proteins extracted from both uninfected and Mtb-infected THP-1 macrophages were subjected to trypsin digestion. We employed an affinity enrichment strategy utilizing anti-diglycine lysine antibody-conjugated agarose beads to generate samples enriched in ubiquitinated peptides. The enriched peptides were analyzed using liquid chromatography-tandem mass spectrometry (LC–MS/MS), and the resulting mass spectrometry data were processed for peptide quantification. To evaluate alterations in protein ubiquitination in response to Mtb infection, we conducted a label-free quantitative proteomic analysis of lysine ubiquitination substrates. All raw ubiquitome read data presented in this study are available *via* ProteomeXchange with identifier PXD063016.

The Principal Component Analysis‌ (PCA) and heat maps indicated a significant alteration in the ubiquitin modification levels of THP-1 macrophage proteins at 7 h post-MTB infection (Figures 1B,C). A total of 828 proteins exhibited upregulated ubiquitination levels, while 790 proteins displayed downregulated ubiquitination levels. In total, 1182 upregulated and 1077 downregulated ubiquitination modification sites were identified (Figure 1D) (Datasheet 1). The average number of upregulated and downregulated ubiquitination modification sites per protein was 1.43 and 1.36, respectively (Figure 1E). Venn diagram analysis revealed that 112 proteins contained both upregulated and downregulated ubiquitination modification sites (Figure 1F). The protein sites with the highest levels of upregulation in ubiquitination included MSR1_K430, ASAH1_K310, PSAP_K348, PSAP_K344, and IFI30_K116, whereas the sites with the greatest downregulation were RPL19_K190, STMN1_K100, GNPTG_K144, ZNF644_K676, and MCM3_K140 (Figure 1G; Table 1).

**TABLE 1:** The list of the top five proteins with the highest and lowest levels of ubiquitination modification upregulation and downregulation.

Protein	Annotation	Site	Fold change
MSR1	Macrophage scavenger receptor types I and II	K430	27.3
ASAH1	Acid ceramidase	K310	23.0
PSAP	Prosaposin	K348	16.6
PSAP	Prosaposin	K344	12.0
IFI30	Gamma-interferon-inducible lysosomal thiol reductase	K116	12.0
RPL19	Large ribosomal subunit protein eL19	K190	−22.3
STMN1	Stathmin	K100	−12.6
GNPTG	N-acetylglucosamine-1-phosphotransferase subunit gamma	K144	−10.2
ZNF644	Zinc finger protein 644	K676	−7.8
MCM3	DNA replication licensing factor MCM3	K140	−7.6

### GO and KEGG analyses of ubiquitinated targets after MTB infection

**FIGURE 2:**
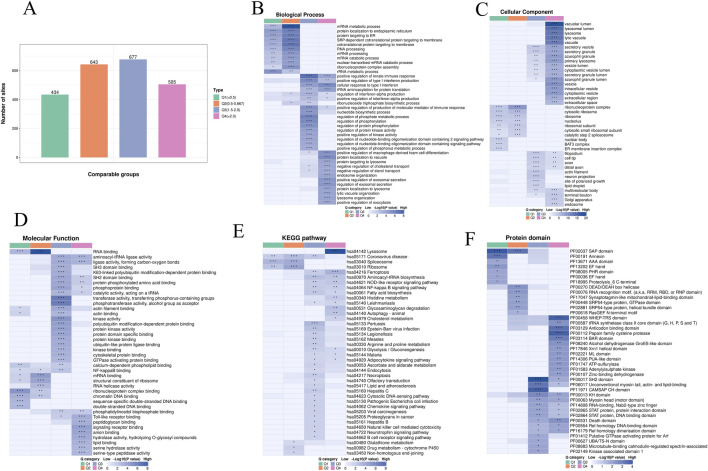
GO and KEGG analyses of ubiquitinated proteins after Mtb infection. **(A)** Fold change distribution of the protein ubiquitination sites. **(B)** Biological process of ubiquitinated proteins by GO classification. **(C)** Cellular component of the ubiquitinated proteins by GO classification. **(D)** Molecular function of the ubiquitinated proteins by GO classification. **(E)** KEGG pathway enrichment of the ubiquitinated proteins. **(F)** Protein domain analysis of the ubiquitinated proteins.

To better understand the changes in the ubiquitome of human macrophages infected by Mtb, we compared the functional similarities and differences among proteins exhibiting varying degrees of differential expression. The proteins were categorized into four groups, designated as Q1 to Q4, representing ubiquitination that are downregulated by 2-fold (Q1), downregulated by 1.5-fold–2-fold (Q2), upregulated by 1.5-fold–2-fold (Q3), and upregulated by 2-fold (Q4), respectively. Enrichment analyses for GO, KEGG, and protein domains analysis were conducted for each group, followed by functional cluster analysis (Figure 2). The number of proteins with ubiquitination that were upregulated by 1.5-fold–2-fold and by 2-fold was 677 and 505, respectively (Figure 2A). In contrast, the number of proteins with ubiquitin modifications that were downregulated by 1.5-fold–2-fold and by 2-fold was 643 and 434, respectively (Figure 2A).

The analysis of biological processes through GO functional clustering revealed that proteins exhibiting significant changes in ubiquitination were associated with various categories, including “mRNA metabolic process,” “protein localization to the endoplasmic reticulum,” “protein targeting to the endoplasmic reticulum,” “signal recognition particle (SRP)-dependent cotranslational protein targeting to the membrane,” “cotranslational protein targeting to the membrane,” and “RNA processing” (Figure 2B). Within the cellular component category, the ubiquitination levels of proteins involved in the “vacuolar lumen,” “lysosomal lumen,” “lysosome,” “lytic vacuole,” “vacuole,” and “secretory vesicle” were significantly enriched (Figure 2C). Regarding molecular functions, proteins that underwent significant changes were predominantly involved in “RNA binding,” “aminoacyl-tRNA ligase activity,” “ligase activity forming carbon-oxygen bonds,” “SH3 domain binding,” “K63-linked polyubiquitin modification-dependent protein binding,” and “SH2 domain binding” (Figure 2D).

To identify the cellular pathways affected by Mtb infection, we conducted a pathway clustering analysis utilizing the KEGG (Figure 2E). The results revealed that the pathways “lysosome,” “coronavirus disease,” “ferroptosis,” “aminoacyl-tRNA biosynthesis,” “NOD-like receptor signaling pathway,” and “NF-κB signaling pathway” were significantly enriched in proteins exhibiting upregulated ubiquitination (Figure 2E). In contrast, the pathways “spliceosome,” “ribosome,” and “coronavirus disease” were primarily enriched in proteins displaying downregulated ubiquitination (Figure 2E). Additionally, we analyzed the protein domains with altered ubiquitination, which indicated that the “WHEP-TRS domain,” “tRNA synthetase class II core domain,” “anticodon binding domain,” “papain family cysteine protease,” and “BAR domain” were the most significantly enriched in proteins with upregulated ubiquitination (Figure 2F). Conversely, the “SAP domain,” “Annexin,” “AAA domain,” “EF hand domain,” and “PHR domain” were the most prominently enriched in proteins with downregulated ubiquitination (Figure 2F).

### Proteomic analyses generate a catalog of ubiquitination targets related to host immune response

**FIGURE 3:**
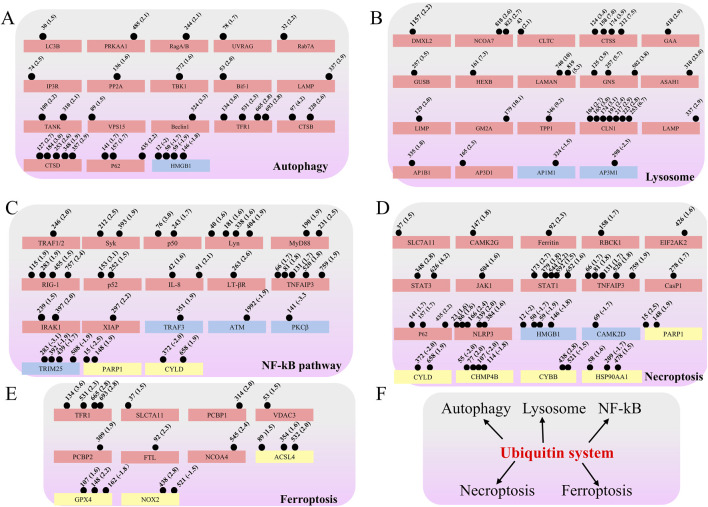
Ubiquitinated proteins involved in many immune response pathways after Mtb infection. Ubiquitinated proteins were classified into four classes: **(A)** autophagy; **(B)** lysosome; **(C)** NF-κB signaling pathway; **(D)** Necroptosis; **(E)** Ferroptosis; **(F)** Model of the regulatory functions of the ubiquitin system in host immune responses after Mtb infection. For **(A–E)**, the red box represents upregulation of ubiquitination, the blue box represents downregulation of ubiquitination, and the yellow box represents the presence of both up- and downregulated sites, the ubiquitination sites of each protein are indicated by small spots, the numbers in parentheses indicate the ratio of upregulation or downregulation of ubiquitination.

Ubiquitination is known to play significant roles in host immune responses (Franco et al., 2017a; Campos et al., 2022; Romagnoli et al., 2023). Therefore, we conducted a search for immune-related proteins among the ubiquitinated proteins. Our findings revealed that proteins associated with “autophagy”, “lysosome”, “NF-κB signaling pathway”, “necroptosis”, and “ferroptosis” (Figures 3A–E) exhibited alterations in ubiquitination. Notably, the NF-κB pathway displayed the highest number of ubiquitination modifications, encompassing a total of 19 proteins, while the ferroptosis pathway recorded the lowest number, with only 10 proteins modified. Among the identified proteins, the lysosome-associated protein CLN1 was found to possess the most ubiquitination sites, totaling seven sites (K104, K165, K174, K191, K217, K223, and K253) that exhibited upregulated ubiquitination. Remarkably, the count of ubiquitination upregulated proteins within the aforementioned immune-associated pathways was 62, significantly exceeding the seven proteins that were downregulated. Furthermore, we identified 12 proteins that exhibited both up- and downregulated ubiquitination sites (Figure 3). These data suggest that ubiquitination in the autophagy, lysosome, NF-κB signaling pathway, necroptosis, and ferroptosis may play a crucial role in immune responses during Mtb infection of the host (Figure 3F).

### Ubiquitination affects many conserved physiological processes

**FIGURE 4:**
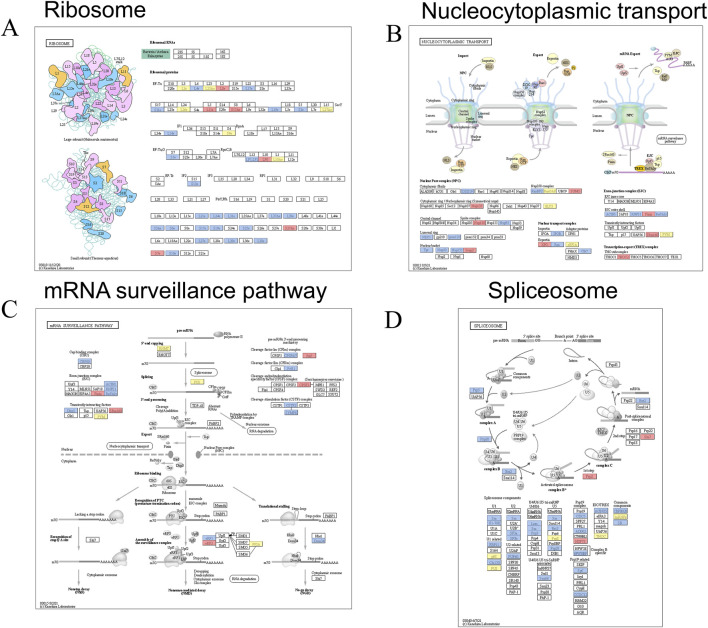
Ubiquitinated proteins involved in many conserved physiological processes after Mtb infection. Ubiquitinated proteins were classified to four classes: **(A)** ribosome; **(B)** Nucleocytoplasmic transport; **(C)** mRNA surveillance pathway; **(D)** Spliceosome; For **(A–D)**, the red box represents upregulation of ubiquitination, the blue box represents downregulation of ubiquitination and the yellow box represents the presence of both up- and downregulated sites.

In addition to the altered ubiquitination of immune-related proteins, our protein ubiquitination analysis revealed that many important proteins associated with physiological processes were similarly enriched. Proteins associated with the “Ribosome,” “Spliceosome,” “Nucleocytoplasmic transport,” and “mRNA surveillance” exhibited changes in ubiquitination (Figure 4). Specifically, a total of 21 proteins displayed upregulated ubiquitination, while 75 proteins exhibited downregulated ubiquitination. Furthermore, 19 proteins possessed both up- and downregulated ubiquitination sites (Figure 4). Notably, our findings indicate that the number of ribosome and spliceosome-associated proteins with downregulated ubiquitination significantly exceeds that of upregulated proteins, with counts of 23 and 27 proteins, respectively (Figures 4A,D).

### Protein interaction networks for the MTB infection triggered ubiquitome

To further analyze the significance and extent of ubiquitination in response to Mtb infection, we generated an interaction network using the Search Tool for Retrieval of Interacting Genes/Proteins (STRING) database (Szklarczyk et al., 2015). The top 50 proteins exhibiting the strongest interactions were selected to construct the protein interaction network (Supplementary Figure S1). Within this network, we identified six proteins positioned at key nodes, namely, STAT1, RPS27A, HDAC1, CREBBP, SNRPD3, and HSP90AA1 (Supplementary Figure S1), their ubiquitination modification sites and the fold changes are STAT1_K173 (2.7 folds increase), STAT1_K544 (2.2 folds increase), STAT1_K592 (1.5 folds increase), STAT1_K652 (1.6 folds increase), STAT1_K379 (1.8 folds increase), RPS27A_K27 (1.5 folds increase), HDAC1_K361 (2.9 folds decrease), CREBBP_K2075 (2.2 folds decrease), SNRPD3_K104 (1.9 folds increase), HSP90AA1_K478 (1.5 folds increase), HSP90AA1_K58 (1.6 folds increase), HSP90AA1_K209 (1.7 folds decrease). Through GO analysis, the proteins enriched in the TOP five pathways were annotated. It was observed that the interacting proteins predominantly participated in processes such as “SPR-dependent co-translational protein targeting to the membrane”, “co-translational protein targeting to the membrane”, “mRNA metabolic processes”, “negative regulation of gene expression”, and “regulation of cellular macromolecule biosynthetic processes” (Supplementary Figure S1).

**FIGURE 5:**
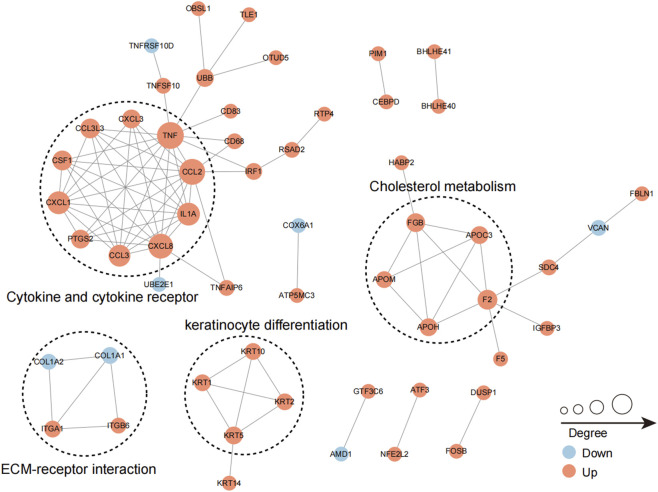
The sub-networks of the protein interaction network. Circles in the figure represent differentially ubiquitinated proteins, different colors represent differentially expressed proteins (blue is downregulated ubiquitinated proteins, red is upregulated ubiquitinated proteins, yellow is ubiquitinated proteins containing upregulated modified sites at the same time), and the size of circles represents the number of proteins interacting with them.

Furthermore, several sub-networks were identified (Figure 5). The most abundant ubiquitinated proteins within the interaction network were those associated with “cytokine and cytokine receptor”. Upon infection with Mtb, several cytokine and cytokine receptor-associated proteins exhibited increased ubiquitination, including CXCL3, TNF, CCL2, IL1A, CXCL8, CCL3, PTGS2, CXCL1, and CSF1 (Figure 5). Similarly, the ubiquitination levels of proteins involved in cholesterol metabolism also increased in response to Mtb infection, specifically APOC3, APOH, and APOM (Figure 5), indicating that cholesterol metabolism is subject to complex regulation during Mtb infection. Additionally, we observed alterations in the ubiquitination levels of proteins related to “ECM-receptor interaction” and “keratinocyte differentiation” following Mtb infection, suggesting the involvement of these two processes in the response to tuberculosis infection (Figure 5).

## Discussion

Although numerous reports have detailed specific instances of ubiquitination in response to Mtb infection, our understanding of the global alterations in the host ubiquitome remains limited. In this study, we utilized high-resolution liquid chromatography-tandem mass spectrometry (LC–MS/MS) combined with an affinity enrichment technique to improve the detection of ubiquitinated peptides. This methodology enabled the large-scale and site-specific quantification of ubiquitination in human macrophages following Mtb infection.

In this study, we identified a total of 1,618 proteins exhibiting altered ubiquitination, including 828 proteins with upregulated ubiquitination levels and 790 proteins with downregulated ubiquitination levels (Figure 1D). The number of ubiquitinated proteins identified in our study was significantly higher than that reported in a previous study, where the authors uncovered the dynamic post-translational modification profiling of Mtb-infected macrophages, identifying only 606 proteins with altered ubiquitination levels (Budzik et al., 2020). The discrepancy in the number of ubiquitinated proteins may be attributed to the different types of macrophages used; the previous study utilized primary murine bone marrow-derived macrophages (BMDMs), whereas our study employed human macrophages. Compared to previous work (Budzik et al., 2020), our research has broadened the list of proteins potentially modified by ubiquitination during tuberculosis infection. These potential targets require validation through additional functional experiments.

Several ubiquitin ligases, including TRIM27, ANAPC2, LNX1, Parkin, and Smurf1, have been reported to play a significant role in restricting Mtb (Manzanillo et al., 2013; Wang et al., 2016; Franco et al., 2017b; Fu et al., 2020; Wang et al., 2020). However, our understanding of the role of this post-translational modification during Mtb infection remains limited. To date, only a few ubiquitinated proteins have been identified, such as NEK6, which is ubiquitinated by LNX1, Rab7, which is ubiquitinated by TRAF6, and MyD88, which is ubiquitinated by Smurf1 (Fu et al., 2020; Peng et al., 2022). In our study, we identified numerous proteins involved in the immune response that are ubiquitinated, including those associated with autophagy, lysosomes, the NF-κB pathway, necroptosis, and ferroptosis (Figure 3). Consistent with previous reports, we also identified ubiquitinated proteins associated with autophagy, such as P62, Rab7, and MyD88 from the NF-κB pathway (Budzik et al., 2020; Peng et al., 2022; Ma et al., 2023). And it appears that these immune-related proteins primarily undergo ubiquitination modifications during the initial stages of infection, whereas their protein expression levels remain relatively unchanged (data not shown).

The immune response-related proteins identified in this study, along with their corresponding ubiquitination sites, provide novel insights for future research on the interaction between Mtb and its host, particularly from an epigenetic perspective. For example, lysosomes, as vital organelles within these cells, play a crucial role in degrading pathogens, which is essential for the host’s protection against Mtb infections (Armstrong and Hart, 1971). We observed alterations in the ubiquitination levels of several lysosomal-related proteins, including lysosomal membrane proteins LAMP and LIMP, as well as lysosomal acid hydrolases like GAA, GUSB, and HEXB, *etc.* The ubiquitination levels of most of these proteins have increased significantly. This suggests that during the process of infection, Mtb may enhance their survival within host cells by inducing ubiquitination modifications on lysosomal components, the specific mechanism of action requires further in-depth exploration.

Interestingly, although studies have reported that necroptosis is favorable for Mtb survival (Zhao et al., 2012; Mohareer et al., 2018), the role of host ubiquitination modifications in this process remains unknown. Our study identified 19 necroptosis-associated proteins with altered levels of ubiquitination modifications, and further investigation is needed to elucidate how these proteins affect Mtb infection (Figure 3). It has been reported that Mtb infection can trigger ferroptosis, thereby promoting its pathogenicity and transmission. Our research identified altered ubiquitination levels in 10 ferroptosis associated proteins. Notably, there are three ubiquitination sites on the key protein glutathione peroxidase 4 (GPX4): upregulation at K107 and K148, and downregulation at K162. According to a recent report, Mtb inhibits GPX4 from inducing ferroptosis by secreting the protein ptpA (Qiang et al., 2023). It has demonstrated that the secreted protein PtpA enters the cell nucleus and interacts with histone H3 at arginine two *via* the target protein arginine methyltransferase 6 (PRMT6). This interaction leads to the asymmetric dimethylation of H3R2 (H3R2me2a), which inhibits the expression of glutathione peroxidase 4 (GPX4) and ultimately induces ferroptosis, thereby enhancing the pathogenicity and transmission of Mtb. In our research, we did not observe any changes in the ubiquitination levels of PRMT6 or H3R2me2a, suggesting that the modification of GPX4 by ubiquitin may be associated with other proteins. While other modes of cell death, such as apoptosis and pyroptosis, have been reported to play a role during Mtb infection, this study did not identify any changes in the ubiquitination levels of the associated proteins (Shi et al., 2014; Joseph et al., 2017; Kelley et al., 2019; Theobald et al., 2021; Chai et al., 2022).

In addition to proteins associated with the immune response to infection, we identified altered levels of ubiquitination in numerous proteins linked to conserved physiological processes, such as ribosomes, spliceosomes, nucleoplasmic transport, and mRNA surveillance pathways (Figure 4). A previous study suggested that vaccinia infection enhances the ubiquitylation of the 40S subunit protein uS10, thereby increasing the burden on ribosome-associated quality control (RQC) pathways during viral propagation (Sundaramoorthy et al., 2021). However, the alteration of ubiquitination levels in ribosomes during Mtb infection has not yet been investigated. Another study reported that spliceosome-mediated alternative splicing of the transcriptome is affected following macrophage infection with Mtb. The regulation of immune-related genes *via* alternative splicing, including IL-4, IL-7, IL-7R, and IL-12R, may significantly influence whether Mtb is activated or remains in a dormant state (Hong et al., 2024). The changes in spliceosome ubiquitination identified in this study offer new insights for further research on the regulation of variable splicing during Mtb infection. In this study, we did not observe any changes in the expression of E3 ubiquitin ligases or deubiquitinating enzymes (data not shown). However, we identified that the ubiquitination levels of some E3 ubiquitin ligases such as RNF213, NEDD4, DTX3L, and MARCHF7 had changed. It is possible that these E3 ubiquitin ligases play a role in the ubiquitination modifications of the proteins we identified.

Through the analysis of the protein interaction network, we identified several key node proteins, including STAT1, RPS27A, HDAC1, CREBBP, SNRPD3, and HSP90AA1 (Supplementary Figure S1). It has been reported that unphosphorylated STAT1 represses apoptosis in macrophages during Mtb infection (Yao et al., 2017), while HDAC1 can suppress IL-12B expression in macrophages during Mtb infection (Chandran et al., 2015). These proteins may play a crucial role in the ubiquitination modifications mediated during the process of Mtb infection. Further research is warranted to explore these proteins in greater depth. Several sub-networks were identified, including “cytokine and cytokine receptor,” “cholesterol metabolism,” “extracellular matrix (ECM)-receptor interaction,” and “keratinocyte differentiation.” It is well established that cytokines can play both positive and negative roles in the development of TB (Domingo-Gonzalez et al., 2016) (Figure 5). Previous studies have indicated that Mtb infection enhances macrophages’ absorption of cholesterol and low-density lipoprotein (LDL) while improving their ability to synthesize cholesterol *de novo* (Chen et al., 2024). The regulatory role of ubiquitination in these processes warrants further investigation.

Although our research has identified many valuable proteins involved in ubiquitination modification during tuberculosis infection, it is important to acknowledge certain limitations of our study. Specifically, we exclusively utilized human macrophages, which may limit the applicability of our findings, as we did not include primary cells or conduct further verification through animal experiments. Additionally, while PR-619 treatment may influence DUB activity, it also resulted in a significant increase in the ubiquitination levels of specific proteins that, despite their original ubiquitination levels not being markedly elevated, are critically important.

In summary, the systematic discovery of ubiquitination targets in Mtb-infected human macrophages has demonstrated the variety and complexity of immune responses involving the ubiquitination system. Beyond its role in the immune response, the ubiquitination system is crucial for regulating conserved physiological processes. This study’s identification of ubiquitination-related proteins and specific ubiquitination sites provides valuable resources for understanding how ubiquitination systems regulate host immune responses during Mtb infection.

## Data Availability

All raw ubiquitome read data presented in this study are available via ProteomeXchange with identifier PXD063016.

## Supplementary material

SUPPLEMENTARY FIGURE S1 Protein interaction network of the ubiquitinated proteins. The protein interaction network was generated with the complete list of ubiquitinated proteins using the STRING database and was visualized by the R package “visNetwork” tool. Circles in the figure represent differentially modified proteins, the proteins were visualized by GO categories, blue is downregulated ubiquitinated proteins, pink is upregulated ubiquitinated proteins, and the size of circles represents the number of proteins interacting with them.
