# Supplementary material for: Insights into the protein ubiquitinome in the host‒pathogen interplay during Mycobacterium tuberculosis infection
Source: Front Mol Biosci. 2025 Aug 26;12:1613454. doi: 10.3389/fmolb.2025.1613454 (PMC12417728; doi:10.3389/fmolb.2025.1613454)
Supplement: Supplementary file 1 [file Image1.pdf]

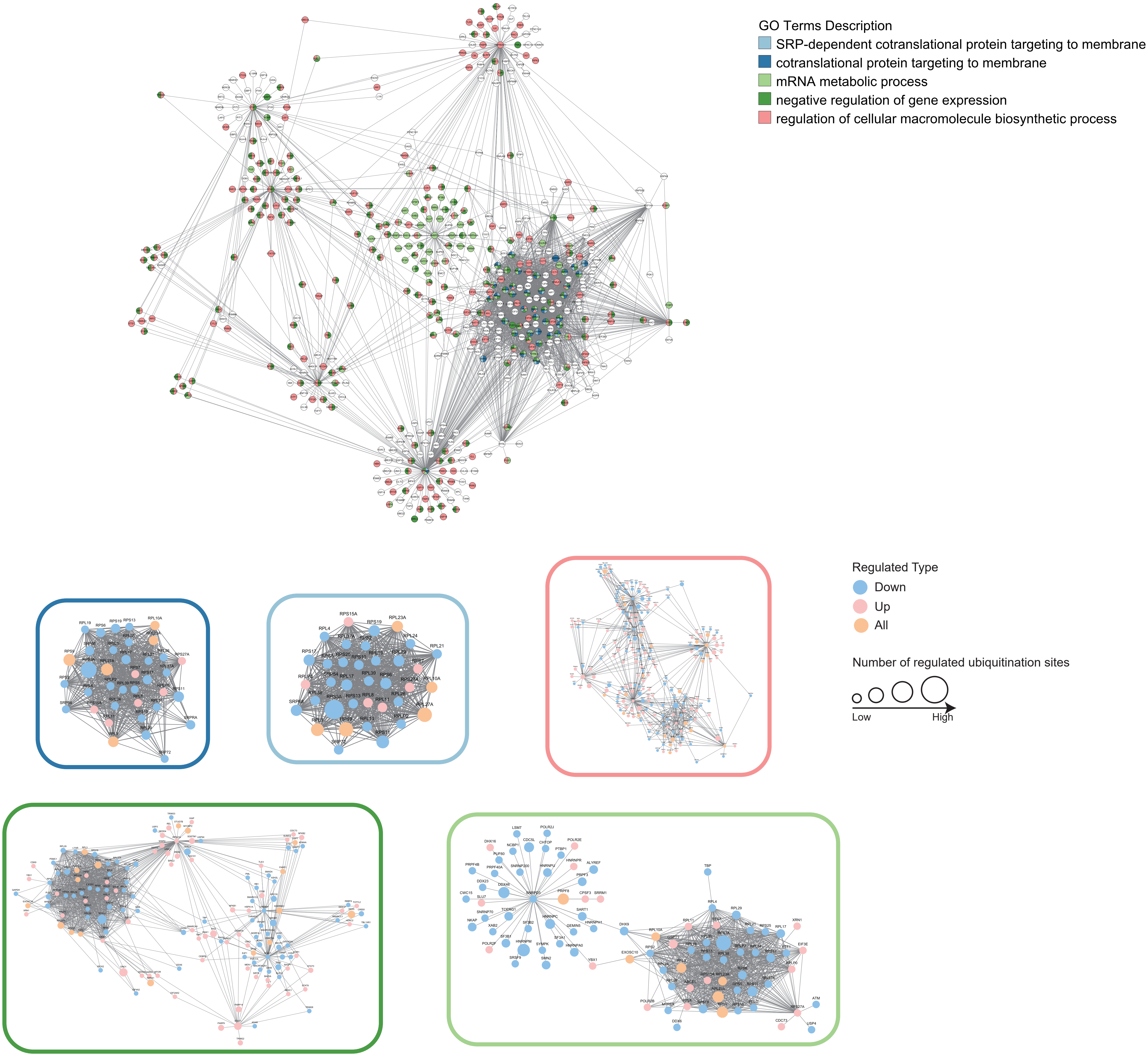
GO Terms Description
SRP-dependent cotranslational protein targeting to membrane
cotranslational protein targeting to membrane
mRNA metabolic process
negative regulation of gene expression
regulation of cellular macromolecule biosynthetic process
Regulated Type
Down
Up
All
Number of regulated ubiquitination sites
Low
High
RPL19
RPS6
RPS19
RPS13
RPL10A
RPL23A
RPS9
RPS27A
RPS2
SRP68
SRPRA
SRP72
RPS15A
RPL23A
RPL4
RPL24
RPL21
RPS17
RPL10A
RPL27A
RPL3
RPS3A
RPS11
LSM7
POLR2J
DHX16
NCBP1
CDC5L
CHTOP
POLR2E
PTBP1
PUF60
PRPF4B
PRPF40A
SNRNP200
HNRNPU
HNRNPR
ALYREF
PRPF3
DDX23
DDX46
CWC15
SLU7
SNRPD3
PRPF8
CPSF3
SRRM1
TBP
TCERG1
SNRNP70
SART1
NKAP
XAB2
SF3B2
HNRNPC
GEMIN5
HNRNPH1
DHX9
POLR2F
SF3B1
SYMPK
SF3A1
HNRNPA0
HNRNPM
SRSF9
SMN2
YBX1
EXOSC10
RPL4
RPL29
XRN1
EIF3E
POLR2B
MTREX
DDX6
CDC73
ATM
USP4
RPS27A
